# Multicenter Surveillance of Capsular Serotypes, Virulence Genes, and Antimicrobial Susceptibilities of *Klebsiella pneumoniae* Causing Bacteremia in Taiwan, 2017–2019

**DOI:** 10.3389/fmicb.2022.783523

**Published:** 2022-03-18

**Authors:** Chun-Hsing Liao, Yu-Tsung Huang, Po-Ren Hsueh

**Affiliations:** ^1^Department of Medicine, Far Eastern Memorial Hospital, New Taipei City, Taiwan; ^2^Department of Medicine, Yang Ming Chiao Tung University, Taipei, Taiwan; ^3^Departments of Laboratory Medicine and Internal Medicine, National Taiwan University Hospital, National Taiwan University College of Medicine, Taipei, Taiwan; ^4^Ph.D. Program for Aging, School of Medicine, China Medical University, Taichung, Taiwan; ^5^Departments of Laboratory Medicine and Internal Medicine, China Medical University Hospital, School of Medicine, China Medical University, Taichung, Taiwan

**Keywords:** hypervirulent *Klebsiella pneumonia*, bacteremia, capsular serotypes, virulence genes, multicenter surveillance, Taiwan

## Abstract

We conducted a longitudinal epidemiological surveillance of hypervirulent *Klebsiella pneumoniae* (hvKP) in Taiwan. Bacteremic KP isolates collected from 16 hospitals in Taiwan between 2017 and 2019 were collected, and the virulent serotypes (K1, K2, K20, K54, and K57), antimicrobial susceptibilities, and virulence genes of these isolates were investigated. During the 3-year period, 1,310 bacteremic KP isolates were collected, of which 27.5% belonged to virulent serotypes, including K1 (*n* = 162), K2 (*n* = 74), K57 (*n* = 56), K54 (*n* = 41), and K20 (*n* = 27). K1 was the most prevalent capsular serotype, with an annual prevalence of 11–15%, and was equally distributed across the four geographic areas. The prevalence of K2 declined significantly in 2019. According to *wzi*-K typing results, 87% of K1 isolates were classified as *wzi*-1. Among K2 isolates, *wzi-*72 (55.4%) and *wzi*-2 (41.9%) were the most common, whereas *wzi*-206 was the most prevalent (48.2%) among K57 isolates, followed by *wzi*-77 (25.0%). *Wzi*-115 accounted for 85.4% of the K54 isolates, whereas *wzi*-95 accounted for 92.6% of K20 isolates. *rmpA* was present in 99.4% of K1, 98.6% of K2, 89.3% of K57, 78.0% of K54, and 84.0% of K20 isolates. *rmpA2* was present in 100% of K1 and 98.6% of K2 isolates but was only present in 64.3% of K57, 58.5% of K54, and 74.1% of K20 isolates. K1 remains the dominant hvKP serotype and is associated with most virulence genes in Taiwan. Further studies are required to elucidate the significance of other virulent serotypes.

## Introduction

*Klebsiella pneumoniae* is an opportunistic pathogen that can cause various infections, including bacteremia, pneumonia, liver abscess, urinary tract infections, and other invasive infections in immunocompromised or frequently healthcare−exposed patients (Choby et al., 2020; Kuo et al., 2021; Zhang et al., 2021). Community-onset hypervirulent *K. pneumoniae* (hvKP) has been reported for several decades, with liver abscess and central nervous system complications as hallmark manifestations (Liu et al., 1986; Fang et al., 2007; Tsai et al., 2008; Russo and Marr, 2019). Capsular serotypes, mainly K1 and K2, are considered virulence markers (Lai et al., 2003; Liao et al., 2011, 2014). However, a previous study using multilocus sequence typing (MLST) showed that all K1 isolates were ST23 or its single locus variants as opposed to K2 isolates with more than 10 ST types (Liao et al., 2014). Recently, several genes in plasmids have been found to be associated with virulence, including *rmpA/rmpA2* (upregulation of capsule expression), *iro* (salmochelin), *iuc* (aerobactin), and *peg-344* (metabolite transporter) (Wu et al., 2009; Bialek-Davenet et al., 2014; Struve et al., 2015; Bulger et al., 2017; Lam et al., 2018a). The definition of hvKP has gradually changed from the presence of specific serotypes to the presence of virulence genes (Bialek-Davenet et al., 2014; Russo and Marr, 2019). A new typing method, *wzi* gene sequencing, has been proposed to determine the capsular types of *Klebsiella* strains because many isolates cannot be typed using the traditional method (Brisse et al., 2013).

There is relatively limited information regarding serotypes other than K1 and K2 that cause invasive infections (Liao et al., 2014; Lee et al., 2016; Turton et al., 2018; Parrott et al., 2021; Wei et al., 2021). We previously showed that K57 is the third most common serotype after K1 and K2, and we found that K57/ST218 is a single locus variant of K1/ST23 and causes liver abscess (Liao et al., 2011, 2014). K57 has also been found in New York, Singapore, and China (Lee et al., 2016; Parrott et al., 2021; Wei et al., 2021). A recent study of 39 K57 isolates from China showed no significant differences in the prevalence of *rmpA* and aerobactin genes between K57 and K1/K2 isolates (Wei et al., 2021). The major ST type of K57 isolates was ST412, followed by ST218 and ST592, and the major *wzi*-K types were 206 and 77. Notably, all three K57 ST types (ST412, ST218, and ST592) could be typed as *wzi-*206 or *wzi-*77 type with *wzi* typing.

Longitudinal epidemiological surveillance of hvKP is sparse in Taiwan, despite the country being the epicenter of hvKP (Huang et al., 2020). Furthermore, information regarding capsular serotypes and their geographic correlations is limited. We prospectively enrolled 213 cases of monomicrobial KP bacteremia in 2017 and compared them with our previous cohort recruited in 2007 from the same institute. We found that the prevalence of K1 was similar (16% in 2017 vs. 19% in 2007), but that of K2 significantly decreased over time (7% in 2017 vs. 17% in 2007, *p* = 0.001) (Huang et al., 2020). To further understand the current epidemiology of hvKP in Taiwan.

We performed an analysis of bacteremic KP isolates collected from 16 hospitals over 3 years (2017–2019), focusing on the prevalence of the proposed invasive serotypes (K1, K2, K20, K54, and K57) and the associated virulence genes.

## Materials and Methods

### Collection of Isolates

From 2017 to 2019, 16 major teaching hospitals in Taiwan, including eight, two, five, and one in the northern, central, southern, and eastern regions, respectively, participated in a nationwide resistance surveillance program (the SMART program) conducted by Taiwan Centers for Disease Control to examine clinically important pathogens (Jean et al., 2018). *Klebsiella pneumoniae* is one of the pathogens collected. In this survey, random and non-duplicate blood samples were collected monthly. Sample collection sites included the emergency department (ED), outpatient department (OPD), general ward, and intensive care unit (ICU). The institutional review board of the National Taiwan University Hospital approved this study (201609066RINB) and waived the need for written informed consent. Patient informed consent was waived because the surveillance program for bacterial isolates presented minimal risk to the subjects.

### Determination of Capsular Serotypes, *Wzi*-K Sequencing, Virulence Genes, and Multilocus Sequence Typing

The genotypes of capsular polysaccharide (CPS) in K1, K2, K20, K54, and K57 isolates were determined using polymerase chain reaction (PCR) (Pan et al., 2008). *Wzi-K* sequencing was performed according to a previously published protocol (Brisse et al., 2013). Virulence genes, including *rmpA, rmpA2, iroB, iucA*, and *peg-344*, were screened using previously reported primers (Yu et al., 2015; Bulger et al., 2017). MLST was performed according to previously described protocols. House-keeping genes, including *gap*A, *inf*B, *mdh*, *pgi*, *pho*E, *rpo*B, and *ton*B, were sequenced and compared with the MLST allele profiles available at http://www.pasteur.fr/mlst.

### Antimicrobial Susceptibility Testing

The minimum inhibitory concentrations (MICs) of antimicrobial agents used against the isolates were determined using a commercial VITEK2^®^ antimicrobial susceptibility system (AST-NB card; bioMérieux, Marcy-l’Étoile, France), as described previously (Jean et al., 2018). The results are presented as resistance categories based on MIC breakpoints recommended by the Clinical and Laboratory Standards Institute in 2020 (Clinical and Laboratory Standards Institute [Clsi]., 2020).

### Statistical Analyses

The extended Mantel–Haenszel chi-square test for linear trends was used to analyze the annual changes in the prevalence of K1, K2, K20, K54, K57, and other isolates. Pearson’s chi-square 2 × 2 (2-sided) test or Fisher’s exact test (2-sided) were used to analyze the sources, geographic distributions, and antimicrobial susceptibility rates. Data were analyzed using the SPSS software (version 15.0; SPSS, Chicago, IL, United States).

## Results

### Overall Virulent Capsular Serotype Distribution, Associated Sources, and Geographic Distribution

**FIGURE 1:**
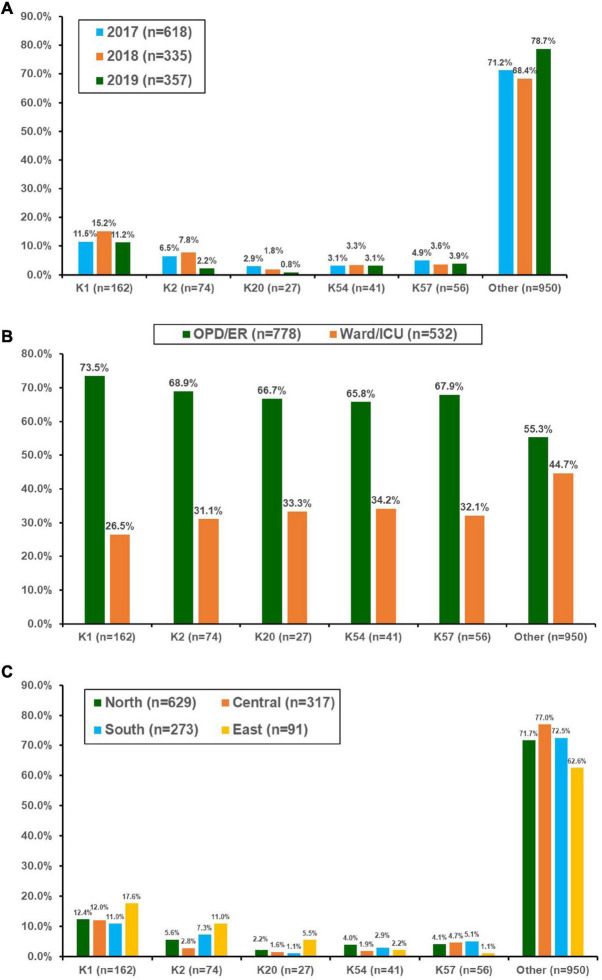
Distribution of the main capsular serotypes of *Klebsiella pneumonia* isolated from patients with bacteremia who were treated at 16 hospitals in Taiwan between 2017 and 2019. **(A)** By year, **(B)** according to location of blood culture collection, and **(C)** by different geographic regions in Taiwan. OPD, outpatient clinics; ER, emergency departments; ICU, intensive care units.

During the 3-year period, 1,310 bacteremic KP isolates were collected (39 isolates were excluded because they were re-identified as other *Klebsiella* species using MALDI-TOF MS). The number 122 of isolates collected in 2017, 2018, and 2019 was 618 (ED/OPD 376, 60.8%), 335 (ED/OPD 198 59.1%), and 357 (ED/OPD 204, 57.1%), respectively. Among these, 27.5% were identified as virulent serotypes, including K1 (*n* = 162), K2 (*n* = 74), K57 (*n* = 56), K54 (*n* = 41), and K20 (*n* = 27). The distribution of these five serotypes is shown in Figure 1A. K1 was the most prevalent capsular serotype, with a prevalence of approximately 11–15%. The prevalence of K2 significantly declined in 2019 (chi-square for linear trend, *p* = 0.01), reaching a level lower than that of K57. When the association between the source of isolates and capsular serotypes was analyzed, K1 was associated with community-acquired infection compared with serotypes other than K1, K2, K20, K54, and K57 (73.5% of isolates from patients at OPD or ED vs. 55.3%, *p* < 0.001) (Figure 1B). K2 was also associated with community-acquired infections (68.9%, *p* = 0.028), but K20, K54, and K57 did not reach statistical significance. In this 3-year surveillance study, K1 was equally distributed across the four geographic areas of Taiwan (Figure 1C). However, the distribution of K2 was significantly different across the four regions (*p* = 0.006).

### Comparison Between Capsular Serotyping and *Wzi*-K Typing

Within the K1 serotype (*n* = 162), *wzi*-K typing revealed that 87% isolates (n = 141) were *wzi*-1, while 2.5% (*n* = 4) were *wzi*-2 (Table 1). Among the K2 isolates, *wzi*-72 (55.4%) and *wzi*-2 (41.9%) were the most common types, whereas *wzi-*206 was the most prevalent type (48.2%) among K57 isolates, followed by *wzi-*77 (25.0%). Notably, *wzi*-2 was found in both K1 and K2 isolates, whereas *wzi-*72 was found in all three CPS genotypes. Furthermore, *wzi*-1, *wzi*-5, *wzi-*83, *wzi*- 149, and *wzi*-208 were observed in both K1 and K57 isolates. In contrast to K2 and K57, which have two major *wzi*-K types, K54 isolates comprised 85.4% *wzi*-115 type, whereas K20 isolates comprised 92.6% *wzi*-95 type.

**TABLE 1:** *Wzi*-K typing of K1, K2, K20, K54, and K57 serotypes of bacteremic *Klebsiella pneumoniae* isolates.

Wzi-K type	Serotype					Total
	K1	K2	K20	K54	K57	
1	141	0	0	0	1	142
2	4	31	0	0	0	35
5	1	0	0	0	1	2
12	0	0	0	0	1	1
14	0	0	0	0	1	1
16	1	0	0	1	0	2
23	0	1	0	0	0	1
24	0	0	0	1	0	1
34	0	0	0	0	1	1
37	0	0	1	0	0	1
39	1	0	0	0	0	1
55	1	0	0	0	0	1
72	1	41	0	0	1	43
77	0	0	0	0	14	14
83	1	0	0	0	1	2
84	0	0	0	0	1	1
95	3	0	25	1	0	29
115	0	0	0	35	0	35
117	0	0	0	2	0	2
128	1	0	0	0	0	1
149	1	0	0	0	1	2
162	0	0	0	1	2	3
173	1	0	0	0	0	1
177	0	0	0	0	1	1
206	0	0	0	0	27	27
208	2	0	0	0	1	3
257	0	1	0	0	0	1
407	0	0	0	0	1	1
427	1	0	0	0	0	1
433	0	0	0	0	1	1
438	1	0	0	0	0	1
461	1	0	0	0	0	1
527	0	0	1	0	0	1
Total	162	74	27	41	56	360

### Comparison Between Capsular Serotyping and Multilocus Sequence Typing

The MLST of these virulent isolates showed marked variations (Table 2). Among the K1 isolates (*n* = 162), ST23 was the dominant type (*n* = 141, 87%). Among the K2 isolates (*n* = 74), ST65, ST86, ST373, and ST 375 were the major types (accounting for 67.6% of the isolates). Among the K57 isolates (*n* = 56), ST592 (23.2%), ST218 (19.6%), and ST412 (17.9%) were the dominant types. ST29 accounted for 71.7% of K54 isolates (*n* = 41), and ST268 (33.3%) and ST1544 (22.2%) were the dominant types among the K20 isolates (*n* = 27).

**TABLE 2:** Multi-locus sequence typing (MLST) of K1, K2, K20, K54, and K57 serotypes of bacteremic *Klebsiella pneumoniae* isolates (only MLST types present in more than one isolate are included).

MLST type	Serotype					Total
	K1	K2	K20	K54	K57	
12	0	0	0	1	1	2
23	134	1	0	1	1	137
25	0	6	0	0	0	6
29	0	0	0	29	0	29
65	0	21	0	0	1	22
86	0	12	0	0	0	12
163	2	0	2	0	0	4
218	0	0	0	0	11	11
268	2	0	9	0	0	11
307	1	0	1	0	1	3
373	0	7	0	0	0	7
375	0	10	0	0	0	10
412	0	0	0	0	10	10
420	0	0	1	1	0	2
524	2	0	0	0	0	2
592	0	0	0	0	13	13
793	3	0	0	0	0	3
801	1	2	0	0	0	3
1,049	2	0	0	0	1	3
1,109	0	1	0	1	1	3
1,479	0	2	0	0	0	2
1,544	0	0	6	0	0	6
2,157	0	1	2	0	0	3
2,180	0	3	0	0	0	3
4,255	0	0	3	0	0	3
5,621	0	0	0	0	2	2

### Distribution of Virulence Genes Among Capsular Serotypes

To further delineate the differences among the major capsular serotypes, we compared the distribution of major virulence genes (Table 3). *rmpA* was present in 99.4% of K1 isolates, 98.6% of K2 isolates, 89.3% of K57 isolates (*p* = 0.001, compared with K1 isolates), 78.0% of K54 isolates (*p* < 0.001), and 81.5% of K20 isolates (*p* < 0.001). *rmpA2* was present in 100% of K1, 98.6% of K2, 64.3% of K57, 58.5% of K54, and 74.1% of K20 isolates. *IroB* was present in 90.1% of K1, 87.8% of K2, 55.4% of K57, 58.5% of K54, and 74.1% of K20 isolates. *IucA* was present in 81.5, 75.7, 69.6, 58.5, and 77.8% of K1, K2, K57, K54, and K20 isolates, respectively. Overall, the differences in the distribution of the virulence genes in K1 and K2 isolates were not significant, but the remaining three serotypes carried fewer virulence genes.

**TABLE 3:** Distribution of virulence genes among capsular serotypes and major *wzi* types.

Serotype				*-rmpA*			*-rmpA2*			-iroB			*-iucA*			*-peg-344*		
	*wzi*			+	%	*P*-value	+	%	*p*-value	+	%	*p*-value	+	%	*p*-value	+	%	*p*-value
K1^R^		162		161	99.4%	−	162	100.0%	−	146	90.1%	−	132	81.5%	−	143	88.3%	−
	*1*	141	87.0%	140	99.3%		141	100.0%		131	92.9%		117	83.0%		127	90.1%	
	2	4	2.5%	4	100.0%		4	100.0%		4	100.0%		4	100.0%		4	100.0%	
K2		74		73	98.6%	0.53	73	98.6%	0.31	65	87.8%	0.65	56	75.7%	0.38	65	87.8%	1.000
	2	31	41.9%	31	100.0%		31	100.0%		29	93.5%		25	80.6%		29	93.5%	
	*72*	41	55.4%	40	97.6%		40	97.6%		35	85.4%		30	73.2%		34	82.9%	
K20		27		22	81.5%	<0.001	20	74.1%	<0.001	23	85.2%	0.50	21	77.8%	0.79	24	88.9%	1.000
	*95*	25	92.6%	21	84.0%		19	76.0%		22	88.0%		20	80.0%		22	88.0%	
	*527*	1	3.7%	1	100.0%		1	100.0%		1	100.0%		1	100.0%		1	100.0%	
K54		41		32	78.0%	<0.001	24	58.5%	<0.001	24	58.5%	<0.001	24	58.5%	0.003	24	58.5%	<0.001
	*115*	35	85.4%	30	85.7%		23	65.7%		23	65.7%		23	65.7%		22	62.9%	
	117	2	2.8%	1	50%		0	0.0%		0	0.0%		0	0.0%		0	0.0%	
K57		56		50	89.3%*	0.001	36	64.3%*	<0.001	31	55.4%*	<0.001	39	69.6%	0.09	43	76.8%*	0.05
	77	14	25.0%	14	100.0%		12	85.7%		11	78.6%		13	92.9%		13	92.9%	
	206	27	48.2%	25	92.6%		22	81.5%		16	59.3%		22	81.5%		26	96.3%	

*Using K1 as the reference serotype.

### Comparison of Antimicrobial Susceptibility in K1, K2, K20, K54, K57, and Other Serotypes

Susceptibility to the five antimicrobial agents tested was higher for the virulent capsular serotype isolates than for the non-virulent capsular serotype isolates (Table 4). Among these, K1 isolates were associated with significantly higher susceptibility. Because of the small number of cases, only some categories of the remaining virulent serotypes reached statistical significance. No decline in annual susceptibility rates was observed in the virulent serotype isolates within the 3- year study period. We further compared the susceptibilities according to the source of patients and serotypes of *K. pneumoniae* (Table 5). Generally, isolates collected from OPD/ER exhibited higher susceptibility rates compared to isolates from general wards/ICUs with both virulent serotypes and other serotypes. The differences were significant for K1 isolates among all classes of antibiotics except ciprofloxacin; however, the differences did not reach statistical significance among the K2 isolates.

**TABLE 4:** Antimicrobial susceptibility of main bacteremic *Klebsiella pneumoniae* serotypes.

Serotype	Year	Number of isolates	Amoxicillin- clavulanate			Ceftazidime			Ciprofloxacin			Ertapenem			Gentamicin		
			S	%	*p*-value	S	%	*p*-value	S	%	*p*-value	S	%	*p*-value	S	%	*p*-value
	2017	71	68	95.8*	<0.001	67	94.4*	<0.001	61	85.9*	<0.001	69	97.2*	0.009	69	97.2*	<0.001
K1	2018	51	51	100.0*	<0.001	51	100.0*	<0.001	50	98.0*	<0.001	51	100.0*	0.011	50	98.0*	<0.001
	2019	40	39	97.5*	0.002	39	97.5*	<0.001	38	95.0*	<0.001	38	95	0.281	39	97.5*	0.002
	2017	40	35	87.5*	0.007	36	90.0*	0.004	34	85.0*	0.002	39	97.5*	0.05	38	95.0*	0.003
K2	2018	26	25	96.2*	0.04	24	92.3	0.05	25	96.2*	0.003	26	100	0.05	24	92.3	0.05
	2019	8	7	87.5	0.68	7	87.5	0.45	6	75	0.72	6	75	0.25	8	100	0.21
	2017	18	17	94.4*	0.02	17	94.4*	0.02	17	94.4*	0.005	18	100	0.148	17	94.4	0.054
K20	2018	6	6	100	0.34	5	83.3	1	3	50	0.412	6	100	1	5	83.3	1
	2019	3	3	100	1	3	100	0.56	3	100	0.555	3	100	1	3	100	1
	2017	19	17	89.5*	0.04	18	94.7*	0.02	16	84.2	0.05	19	100	0.15	19	100.0*	0.006
K54	2018	11	10	90.9	0.46	10	90.9	0.3	11	100.0*	0.02	11	100	0.37	10	90.9	0.47
	2019	11	9	81.8	1	9	81.8	0.73	10	90.9	0.1	11	100	0.62	10	90.9	0.47
	2017	30	23	76.7	0.24	24	80	0.16	24	80.0*	0.03	27	90	0.78	26	86.7	0.14
K57	2018	12	11	91.7	0.47	11	91.7	0.3	9	75	0.76	12	100	0.37	11	91.7	0.3
	2019	14	13	92.9	0.2	13	92.9	0.12	13	92.9*	0.04	14	100	0.38	13	92.9	0.2
	2017	440	289	65.7		297	67.5	-	263	59.8	-	380	86.4		326	74.1	
Others’^*,†^”	2018	229	177	77.3		170	74.2	-	152	66.4	-	199	86.9		172	75.1	
	2019	281	211	75.1		200	71.2	-	179	63.7	-	248	88.3		213	75.8	

*S, susceptible.*

*Represents p < 0.05.

*†Serotypes other than K1, K2, K20, K54, and K57 as reference.*

**TABLE 5:** Antimicrobial susceptibility according to the source of patients and bacteremic *Klebsiella pneumoniae* serotypes.

Serotype	Source	Number of isolates	Amoxicillin- clavulanate			Ceftazidime			Ciprofloxacin			Ertapenem			Gentamicin		
			S	%	*p*-value	S	%	*p*-value	S	%	*p*-value	S	%	*p*-value	S	%	*p*-value
K1	OPD/ER	119	119	100	0.005	118	99.2	0.018	112	94.1	0.95	119	100	0.005	118	99.2	0.06
	Ward/ICU	43	39	90.7		39	90.7		37	86		39	90.7		40	93	
	OPD/ER	51	48	94.1	0.19	48	94.1	0.19	45	88.2	0.88	50	98	0.23	49	96.1	0.58
K2	Ward/ICU	23	19	82.6		19	82.6		20	87		21	91.3		21	91.3	
	OPD/ER	18	17	94.4	1	17	94.4	1	15	83.3	0.7	18	100	1	17	94.4	1
K20	Ward/ICU	9	9	100		8	88.9		8	88.9		9	100		8	88.9	
	OPD/ER	27	25	92.6	0.31	26	96.3	0.1	24	88.9	0.68	27	100	1	26	96.3	1
K54	Ward/ICU	14	11	78.6		11	78.6		13	92.9		14	100		13	92.9	
	OPD/ER	38	34	89.5	0.13	35	92.1	0.1	35	92.1	0.005	38	100	0.03	37	97.4	0.02
K57	Ward/ICU	18	13	72.2		13	72.2		11	61.1		15	83.3		13	72.2	
	OPD/ER	525	435	82.9	<0.001	440	83.7	<0.001	388	73.9	<0.001	499	95	<0.001	447	85.1	< 0.001
Others	Ward/ICU	425	242	56.9		227	53.4		206	48.5		328	77.2		264	62.1	

## Discussion

HvKP strains have been reported for over 30 years (Liu et al., 1986; Choby et al., 2020). The definition of hvKP has gradually changed, from the ability to cause liver abscess *in vivo* and low LD50 for mouse lethality, to the characterized virulent serotypes, mainly K1 or K2, and nowadays the presence of virulence genes (Russo and Marr, 2019). However, longitudinal epidemiological surveillance data for hvKP are limited. In this 3-year prospective, multicenter surveillance study of bacteremic KP isolates, K1 remained the dominant hvKP serotype and was associated with most virulence genes. The prevalence of K2 significantly decreased in 2019, whereas that of K57 remained unchanged.

Community-onset KP bacteremia is a hallmark of hvKP. However, infections caused by virulent serotypes have also been observed in hospitals. Similar to our previous study, the present study showed that K1 and K2 serotypes were strongly associated with community-onset infections, although the prevalence of K1 serotype found in hospital-onset bacteremia was higher (26.5% compared with 12.2% in our previous observational study) (Liao et al., 2011). Notably, K57 was also associated with community-onset infection, but the difference was not statistically significant. Although community KP bacteremia with liver abscess has been observed across the Taiwanese island (Tsai et al., 2008), simultaneous comparisons among hospitals from different geographical locations are limited. We demonstrated that K1 was prevalent across all four geographic regions (11.0–17.6%). The prevalence of K2 was low in central Taiwan (2.5%), but high in eastern Taiwan (11.0%). The exact reason for this difference remains unknown. Intestinal carriage of virulent serotypes has been reported in Taiwan, Hong Kong, Singapore, and Korea (Siu et al., 2011; Chung et al., 2013; Huang et al., 2018). A recent study of samples from Madagascar, Cambodia, and Senegal showed a very different pattern of KP carriage (Huynh et al., 2020). Although the carriage rate was high (55.9%), the common virulent ST types, including K1/ST23, K2/ST86, K2/ST65, K57/ST218, and K20/ST268, were not observed in that study, which might explain the different clinical presentations. Further research is required to determine whether these virulent serotypes can be detected in food in Taiwan, resulting in intestinal carriage (Davis and Price, 2016; Hartantyo et al., 2020).

In the present study, we performed *wzi* typing and MLST for the five virulent serotypes. An overlap was observed between CPS serotyping and *wzi* typing. Previously, most K1 isolates belonged to ST23 (Liao et al., 2014), but *wzi* typing can further classify K1 isolates into more than 10 *wzi*-K types. In contrast, K2 isolates were classified into more than 10 ST types in the aforementioned report, but only four *wzi* types were identified in the present study. Moreover, there was a significant overlap in *wzi* typing between the K1 and K57 isolates. Interestingly, ST11 (associated with carbapenem resistance, Lee et al., 2012), ST23 (associated with K1), and ST65/ST86 (associated with K2, Liao et al., 2014), were found among the K57 isolates. The complicated association among serotypes, ST types, and *wzi* typing suggests that a single typing method might be insufficient to classify virulent strains. Furthermore, although K1 is considered a prototype of a virulent KP strain, the exact evolution of virulent KP remains elusive.

Information regarding K54 and K20 capsular types is limited. A United Kingdom reference laboratory previously showed that 15 of 31 isolates of K54 isolates were clonal group 29 and carried *rmpA*, *rmpA2*, *iutA*, and *iroD* genes on virulence plasmids (Turton et al., 2018). Comparative chromosomal analysis showed that K54 shared genes with K1/ST23. In this surveillance study, K54 was found in all regions of Taiwan and was the fourth most common virulent serotype. ST29 comprised 71.7% of K54 isolates and carried virulence genes, but its prevalence was lower than that of K1. *Wzi-K* typing showed that 85.4% isolates were *wzi*-115. An Iranian study identified K54 as the dominant type among the typeable isolates, followed by K20 (Hasani et al., 2020). *wcaG* was associated with K54-positive isolates, whereas *rmpA* was associated with K20 isolates. Another study from Taiwan showed that K1 was the predominant serotype among PCR-typeable isolates (41.3%), followed by K2, K20, K5, K54, and K57, but it included only four K54 isolates and six K20 isolates (Chen et al., 2020).

Several important virulence genes associated with hvKP have been identified over the past decade, particularly in virulent plasmids (Bialek-Davenet et al., 2014; Struve et al., 2015; Bulger et al., 2017; Lam et al., 2018b). Performing whole-genome sequence analysis in clinical practice is impractical; thus, screening for important virulence genes is an alternative approach. In a recent publication from New York, virulent gene screening showed that hvKP is associated with hepatobiliary disease and carries a higher mortality risk, contrary to the findings of previous studies in hvKP-endemic areas (Parrott et al., 2021). In the present study, we examined the presence of *rmpA, rmpA2, iroB, iucA*, and *peg-344* among five virulent serotype isolates. Overall, the prevalence of virulence genes in K1 and K2 isolates was comparable; however, K20, K54, and K57 isolates carried fewer virulence genes. The analysis of virulence genes showed that K1 and K2 were still more virulent than K57. The association between virulence genes and capsular serotypes can vary significantly in different countries. The K1 and K2 serotypes isolated from non-hvKP-endemic regions might not carry virulent plasmids. Overall, virulent serotypes were associated with higher antimicrobial susceptibility than the remaining isolates possibly because virulent serotypes were more prevalent in the community setting.

This surveillance study had some limitations. First, information regarding the focus of bacteremia was not collected. Second, *in vitro* hypervirulence tests, such as LD50 for mouse lethality or high-grade serum resistance, were not performed. Third, we did not examine all known virulent serotypes (such as K4 and K5) for comparison, therefore, the study could not exclude the possibility of the absence of hypervirulence characteristics of the isolates with virulence genes detected, particularly for non-K1/K2 isolates. Fourth, food might play an important role in the intestinal carriage of KP, but relevant information in Taiwan is lacking (Davis and Price, 2016; Hartantyo et al., 2020; Lin et al., 2021).

## Summary

K1 remains the dominant hvKP serotype and is associated with the most virulence genes in Taiwan. Further research is necessary to confirm whether the decline in K2 serotype is transient. K57 is the third most common virulent serotype and is associated with community-acquired infections, although it harbors significantly fewer virulence genes.

## Data Availability Statement

The original contributions presented in the study are included in the article/supplementary material, further inquiries can be directed to the corresponding author/s.

## Author Contributions

C-HL and Y-TH conceived and designed the experiments, performed the experiments, analyzed the data, and wrote the manuscript. C-HL, Y-TH, and P-RH read and approved the final version of the manuscript. All authors contributed to the article and approved the submitted version.

## Conflict of Interest

The authors declare that the research was conducted in the absence of any commercial or financial relationships that could be construed as a potential conflict of interest.

## Publisher’s Note

All claims expressed in this article are solely those of the authors and do not necessarily represent those of their affiliated organizations, or those of the publisher, the editors and the reviewers. Any product that may be evaluated in this article, or claim that may be made by its manufacturer, is not guaranteed or endorsed by the publisher.
